# Socioeconomic inequalities of pregnancy termination among reproductive age women in Bangladesh: a decomposition analysis using demographic and health survey

**DOI:** 10.1186/s13104-024-06935-0

**Published:** 2024-10-14

**Authors:** Md. Aslam Hossain, A. M. Mujahidul Islam, Mortuja Mahamud Tohan, Md. Ashfikur Rahman

**Affiliations:** 1 Health Research Group, Department of Statistics, University of Rajshahi, Rajshahi, 6205 Bangladesh; 2 Bangladesh Bureau of Statistics (BBS), Ministry of Planning, Khulna, 9220 Bangladesh; 3 Development Studies Discipline, Social Science School, Khulna University, Khulna, 9208 Bangladesh; 4 School of General Education , Brac University, Dhaka, Bangladesh

**Keywords:** Pregnancy termination, Reproductive age, Inequality, Women, Bangladesh

## Abstract

**Objectives:**

Undergoing women of pregnancy termination (PT) significantly faces the problem of physical and mental health. This study aims to assess the prevalence and socioeconomic disparity factors of PT in Bangladesh. This study analyzed data from the 2017–18 Bangladesh Demographic and Health Survey. Researchers employed chi-square tests to identify relationships between categorical variables and logistic regression to pinpoint factors associated with PT. To assess the socioeconomic variation of PT, the analysis utilized concentration curves, concentration indices, and decomposition techniques.

**Results:**

The study found that 21.0% of reproductive-aged women in Bangladesh had ever terminated a pregnancy. Our study revealed that women from Chittagong and Sylhet regions, with wealthier backgrounds, aged 30 or older, employed in business, taking short birth intervals, and whose husband/partner was 35 or older, were more likely to have had a pregnancy termination with statistical significance (*p* < 0.05). Besides, concentration curves showed a higher prevalence of PT among wealthier women (CCI = 0.029, *p* < 0.001). Decomposition of this inequality revealed that a woman’s wealth status was the largest contributor (74.98%) to the observed disparities, followed by exposure to mass media (41.82%), place of residence (34.35%), occupation (24.81%), and preceding birth interval (6.53%). Our study recommended that, in mitigating the above disparities, we should foster open discussions about underlying factors contributing to PT in Bangladesh.

## Background

Pregnancy termination (PT) is a complex issue with far-reaching consequences for global reproductive health. Particularly when performed unsafely, PT can result in complications such as bleeding, infection, and uterine perforation, which can have major health effects [1, 2]. Worldwide, unsafe PT practices are to blame for between 4.7% and 13.2% of maternal deaths annually [3]. According to the Bangladesh Maternal Mortality Survey 2011, the 1% mortality rate of mother’s accounts for unsafe abortions. Despite a rise in modern contraceptive use, a Bangladesh survey revealed that nearly half (48%) of all pregnancies in the country, approximately 2.8 million, were unintended [4]. It intersects with various aspects of reproductive healthcare, encompassing public health considerations, legal frameworks, social norms, and cultural beliefs [5]. “PT performed by unskilled personnel or in substandard conditions” is the report provided by the World Health Organization (WHO) for unsafe PT [6]. In developing nations, 97% of unsafe abortions take place, comprising roughly 45% of the global total. Annually, an estimated 73 million induced PTs occur worldwide. Of all unwanted pregnancies, three in ten (29%) culminate in induced abortion [7]. The WHO notes that women in resource-constrained countries face higher risks of lifetime pregnancies and pregnancy-related deaths compared to those in high-income nations [8]. Developed regions experience approximately 30 deaths per 100,000 unsafe abortions, while underdeveloped nations see a staggering 220 deaths per 100,000 unsafe abortions. Bangladesh, with its dense population of 16.3 million, records 1,194,000 intentional PT, equivalent to 29 per 1,000 women aged 15–49 [9].

A PT is one of the primary proximal factors of fertility and birth rates, along with marital status and the use and necessity of contraceptives [10]. The primary risk factors for PT include age, marital status, the number of living offspring, and educational attainment [11]. This study in Bangladesh examines socioeconomic factors in PT among women of reproductive age. Bangladesh’s diverse population and sociocultural context offer a valuable opportunity to investigate these inequalities. It fills a research gap and uses decomposition analysis to uncover influences on PT rates among different socioeconomic groups. The aim is to inform public health policies and interventions by assessing PT prevalence and identifying key socioeconomic contributors to disparities.

## Methods

### Data source and study population

This research leverages a cross-sectional design, analyzing secondary data from the 2017–2018 Bangladesh Demographic and Health Survey (BDHS). The BDHS employs a two-stage stratified cluster sampling method, selecting enumeration areas (EAs) in the first stage and households within each EA in the second stage. The 2017–2018 BDHS targeted 20,250 households across 425 rural and 250 urban EAs, aiming to interview approximately 20,100 married women aged 15–49 years. Our analysis focuses on the women’s data file from this survey, which offers insights into sample structure and implementation methods [12].

### Outcome variable

PT was the outcome variable of this study. It was classified into two classes such as (1) PT (no, code, 0) and PT (Yes, code, 1). The survey included the question, “Ever had a terminated pregnancy?”

### Exposure variable

This study investigated a comprehensive set of covariates to understand their potential association with PT decisions in Bangladesh, drawing on insights from prior research. This study explored geographic factors (administrative region and residence type), sociodemographic characteristics (religion, sex of household head, media exposure, wealth index), and various aspects of the woman’s background (age, height, age at first intercourse, occupation, experience of wife beating). Reproductive history (contraceptive use, total number of children ever born, preceding birth interval) and characteristics of the woman’s husband/partner (age, occupation) were also analyzed.

### Statistical analysis

Statistical analyses were conducted using Stata v14.2, incorporating the ‘Svy’ command to accommodate the complex survey design and sampling weights. Earlier analysis and data cleaning procedures addressed missing values and variable recoding. Descriptive statistics were utilized to assess PT prevalence among women aged 15–49 years. Categorical variables were compared using Pearson chi-square. Logistic regression models calculated unadjusted and adjusted odds ratios with 95% confidence intervals to evaluate associations between independent variables and PT. The final model underwent a multicollinearity assessment. All tests were two-sided, with significance set at p-value < 0.05.

### Inequality measurement

Concentration curves (CCs) and concentration indices (CCIs) were utilized to explore and quantify inequality in pregnancy termination (PT) across various socioeconomic characteristics. The CC itself was constructed by plotting the cumulative proportion of women, ranked by wealth index against the cumulative prevalence of PT. A 45-degree line from the origin represents perfect equality. If the CC falls on this line, it suggests PT is evenly distributed across wealth groups. However, a CC below 45-degree line of equality indicates that wealthier (poorer) women are more (less) likely to PT. The further the CC deviates from the equality line, the greater the degree of inequality. Mathematically, the CCI is expressed as:

$$\:CCI=\frac{2}{\mu\:}{cov}({y}_{i},{R}_{i})$$

Where CCI denotes the concentration index; $\:{y}_{i}$​ represents the index of the outcome variable; R signifies the fractional rank of individual i in the socioeconomic position distribution; *µ* indicates the mean of the outcome variable in the sample, and cov represents covariance [13]. The index ranges from − 1 to 1 for unbounded variables. A negative CCI indicates higher concentration among the disadvantaged, while a positive CCI suggests higher concentration among the advantaged. In essence, a CCI of 1 implies all health variables are concentrated with the richest individual, whereas − 1 indicates all health variables are concentrated with the poorest individual. The STATA commands Lorenz [14] and conindex [15] were used to generate the concentration curve and compute the concentration index, respectively.

### Decomposition of the concentration index

Wagstaff decomposition analysis was used to break down the CCI, revealing how individual factors contribute to income-related inequalities. For any linear additive regression model of health outcome (y) [13], such as:

$$\:y=\alpha\:+{\sum\:}_{k}{\beta\:}_{k}{x}_{k}+\epsilon\:$$

Where y is a health outcome variable (socioeconomic inequality of PT in this case), $\:{x}_{k}$stands for a set of socioeconomic determinants influencing the health outcome, α denotes an intercept, $\:{\beta\:}_{k}$represents the coefficient associated with $\:{x}_{k}$. The residual term (ε) reflects the residual inequality in PT used by systematic variations across income groups $\:{x}_{k}$, ideally approaching zero in a well-specified model [16]. The contribution of each determinant to overall wealth-related inequality is calculated as the product of its elasticity and the degree of wealth-related inequality in the CCI of determinant. The “elasticity” column indicates the change in the dependent variable resulting from a one-unit change in the explanatory variables. The sign of the elasticity (+ or -) indicates a corresponding increase or decrease in PT use with a positive change in the determinants [17].

## Results

### Background characteristics

Survey conducted in this study of 20,127 women in Bangladesh revealed a national prevalence of pregnancy termination (PT) at 21%. Women over 30 years old (68%), those living in urban areas (over 60%), and residents of the Dhaka region (over 14%) had higher rates of PT. Early initiation of sexual activity (over 73%) and frequent mass media exposure (around 67%) also emerged as factors associated with increased PT. (**see** Table 1).

Table 2 shows that terminated pregnancy was significantly (*p* < 0.001) associated with the administrative region, place of residence, wealth index, women’s current age, women’s occupation, preceding birth interval, children ever born, husband/partner’s current age having a chance of being terminated pregnancy for the women in the study time. Again, the chance of having a PT is significantly (*p* = 0.010) associated with women who have connected with mass media exposure. Besides, PT was significantly (*p* = 0.004) higher among women who begun first sexual intercourse at age < 18 years than counterparts.

**Table 1:** Bivariate association test between PT and explanatory variables

Study variables	Total, N (%)	Pregnancy Termination		p-value	Weighted Prevalence (95% CI)
		No, N (%)	Yes, N (%)		
**Administrative region**				< 0.001	
Barisal	2154 (10.7)	1,666 (10.5)	488 (11.5)		22.10 (19.77–24.62)
Chittagong	2905 (14.4)	2,423 (15.2)	482 (11.4)		16.47 (15.29–17.71)
Dhaka	2974 (14.8)	2,364 (14.9)	610 (14.4)		20.24(19.16–21.36)
Khulna	2630 (13.1)	2,050 (12.9)	580 (13.7)		21.13(19.52–22.83)
Mymensingh	2167 (10.8)	1,730 (10.8)	437 (10.3)		20.52 (18.58–22.61)
Rajshahi	2576 (12.8)	2,015 (12.7)	561 (13.3)		21.00(19.54–22.55)
Rangpur	2492 (12.4)	1,939 (12.2)	553 (13.1)		21.10(19.51–22.79)
Sylhet	2,229 (11.1)	1,714 (10.8)	515 (12.2)		22.69 (20.41–25.16)
**Place of residence**				< 0.001	
Urban	12,753 (63.4)	10,199 (64.1)	2554 (60.4)		21.51(20.46–22.59)
Rural	7374 (36.6)	5702 (35.9)	1672 (39.6)		19.60(18.96–20.25)
**Religion**				0.085	
Islam	18,136 (90.1)	14,293 (89.9)	3843 (90.9)		20.27(19.70-20.87)
Hinduism	1861 (9.2)	1499 (9.4)	362 (8.6)		18.87(17.09–20.79)
Others	130 (0.6)	109 (0.7)	21 (0.5)		18.33(12.91–25.37)
**Sex of household head**				0.449	
Male	17,273 (85.8)	13,631 (85.7)	3,642 (86.2)		20.25(19.66–20.86)
Female	2,854 (14.2)	2,270 (14.3)	584 (13.8)		19.52(18.13–20.99)
**Mass media exposure**				0.010	
No	6,981 (34.7)	5586 (35.2)	1395 (33.1)		19.54(18.62–20.49)
Yes	13,146 (65.3)	10,315 (64.8)	2831 (66.9)		20.45(19.78–21.15)
**Wealth index**				< 0.001	
Poorest	3,826 (19.0)	3,122 (19.6)	704 (16.7)		18.38(17.17–19.66)
Poorer	3,833 (19.1)	3,039 (19.1)	794 (18.8)		20.26 (19.03–21.54)
Middle	3,883 (19.3)	3,067 (19.3)	816 (19.3)		19.91(18.71–21.16)
Richer	4,088 (20.3)	3,232 (20.3)	856 (20.3)		19.59(18.41–20.82)
Richest	4,497 (22.3)	3,441 (21.6)	1,056 (24.9)		22.40(21.16–23.69)
**Women’s current age**				< 0.001	
< 30 **years**	9037 (44.9)	7691 (48.4)	1346 (31.8)		14.19 (13.49–14.92)
≥ 30 **years**	11,090 (55.1)	8210 (51.6)	288 (68.2)		25.15 (24.34–25.97)
**Women’s height**				0.257	
Below average (< 164 cm)	19,626 (97.5)	15,495 (97.4)	4131 (97.7)		20.19(19.64–20.76)
Average or above (≥ 164 cm)	501 (2.5)	406 (2.5)	95 (2.3)		18.12(15.01–21.72)
**Women age at first intercourse**				0.004	
non-adolescence	14,399 (71.5)	11,301 (71.1)	3098 (73.3)		20.71 (20.06–21.37)
adolescence	5728 (28.5)	4600 (28.9)	1128 (26.7)		18.62(17.61–19.67)
**Women occupation**				< 0.001	
Homemakers	16,515 (82.1)	13,157 (82.8)	3358 (79.5)		19.47(18.57–20.08)
Labour	1402 (7.0)	1091 (6.8)	311 (7.4)		21.86(19.77–24.09)
business	1761 (8.7)	1320 (8.3)	441 (10.4)		23.84(21.88–25.92)
Service Holder	442 (2.2)	328 (2.1)	114 (2.7)		26.15(21.99–30.78)
**Wife beating**				0.109	
low	16,131 (80.2)	12,781 (80.4)	3350 (79.3)		19.85(19.24–20.48)
high	3996 (19.8)	3120 (19.6)	876 (20.7)		21.27(20.04–22.54)
**Contraceptive usage**				0.727	
No	8282 (41.1)	6553 (41.2)	1729 (40.9)		19.83(18.99–20.69)
Yes	11,845 (58.8)	9348 (58.8)	2497 (59.1)		20.37(19.64–21.11)
**Total children ever born**				< 0.001	
≤ 2	12,067 (59.9)	9802 (61.6)	2265 (53.6)		17.76(17.08–18.45)
≥ 3	8060 (40.1)	6099 (38.4)	1961 (46.4)		23.68 (22.76–24.61)
**Preceding birth interval (Y**ears)				< 0.001	
< 2	2109 (15.3)	1686 (16.1)	423 (12.9)		19.05 (17.42–20.79)
2–4	4424 (32.1)	3390 (32.3)	1034 (31.5)		21.99 (20.80-23.24)
> 4	7232 (52.5)	5409 (51.6)	1823 (55.6)		24.74 (23.76–25.75)
**Husband/partner’s current age (Years)**				< 0.001	
< 30	3405 (16.9)	3028 (19.1)	377 (8.9)		10.75 (9.77–11.81)
30–34	2859 (14.2)	2340 (14.7)	519 (12.3)		16.83 (15.51–18.24)
35–39	3389 (16.8)	2657 (16.7)	732 (17.3)		20.25 (18.93–21.64)
≥ 40	10,474 (52.1)	7876 (49.5)	2598 (61.8)		24.25 (23.43–25.09)
**Husband/partner’s occupation**				0.544	
Unemployed	409 (2.2)	318 (2.1)	91 (2.3)		21.85 (18.07–26.18)
Farmer/Labour	8684 (46.1)	6874 (46.3)	1810 (45.1)		20.42 (19.60-21.26)
Business	8533 (45.2)	6696 (45.1)	1837 (45.8)		20.24 (19.39–21.12)
Service Holder	1235 (6.5)	961 (6.5)	274 (6.8)		20.61 (18.31–23.12)

Table 2 presents that woman residing in the Chittagong administrative region of Bangladesh had 0.74 times higher odds of PT compared to those in the Khulna region (AOR = 0.74, 95% CI: 0.63–0.86, *p* < 0.001), while women in Sylhet had 1.19 times higher odds (AOR = 1.19, 95% CI: 1.02–1.41, *p* = 0.031). The likelihood of PT increased with rising wealth index among reproductive women, with the poorest and richest socioeconomic statuses associated with 0.87 (AOR = 0.87, 95% CI: 0.75 to 0.98, *p* = 0.031) and 1.24 (AOR = 1.24, 95% CI: 1.08 to 1.42, *p* = 0.002) times higher odds, respectively, compared to those in the middle wealth status. Women aged ≥ 30 years had 1.57 times higher odds of PT compared to those aged < 30 years (AOR = 1.57, 95% CI: 1.36 to 1.79, *p* < 0.001). Those involved in business occupations had 1.41 times higher odds (AOR = 1.41, 95% CI: 1.23 to 1.62, *p* < 0.001), and those with short birth intervals had 18% higher odds (AOR = 0.82, 95% CI: 0.72 to 0.93, *p* = 0.002) compared to those with optimal birth intervals. Additionally, the risk of PT was 1.39 times higher (AOR = 1.39, 95% CI: 1.08 to 1.79, *p* = 0.011) when the husband/partner’s age was ≥ 35 years compared to < 30 years.

**Table 2:** The Socio-demographic influence factors on pregnancy termination among reproductive women in Bangladesh

Study variable	Unadjusted		Adjusted	
	UOR(95% CI)	p-value	AOR (95% CI)	p-value
**Administrative Region**				
Barisal	1.03 (0.90–1.19)	0.619	1.11 (0.94–1.30)	0.228
Chittagong	0.71 (0.61–0.81)	< 0.001	0.74 (0.63–0.86)	< 0.001
Dhaka	0.91 (0.80–1.04)	0.159	0.95 (0.81–1.11)	0.521
Khulna	Reference		Reference	
Mymensingh	0.89 (0.77–1.03)	0.112	0.98 (0.84–1.16)	0.868
Rajshahi	0.98 (0.86–1.12)	0.810	1.04 (0.89–1.21)	0.644
Rangpur	1.01 (0.88–1.15)	0.905	1.12 (0.95–1.31)	0.162
Sylhet	1.06 (0.93–1.21)	0.382	1.19 (1.02–1.41)	0.031
**Place of residence**				
Rural	Reference		Reference	
Urban	1.17 (1.09–1.25)	< 0.001	1.07 (0.98–1.18)	0.145
**Mass media exposure**				
No	Reference		Reference	
Yes	1.09 (1.02–1.18)	0.010	1.04 (0.94–1.14)	0.407
**Wealth index**				
Poorest	0.85 (0.75–0.95)	0.004	0.87 (0.75–0.98)	0.031
Poorer	0.98 (0.88–1.09)	0.746	1.01 (0.88–1.14)	0.959
Middle	Reference		Reference	
Richer	0.99 (0.89–1.11)	0.934	1.05 (0.92–1.19)	0.450
Richest	1.15 (1.04–1.28)	0.007	1.24 (1.08–1.42)	0.002
**Women current age (Years)**				
< 30	Reference		Reference	
≥ 30	2.01 (1.86–2.15)	< 0.001	1.57 (1.36–1.79)	< 0.001
**Women age at first intercourse**				
Non-adolescence	1.12 (1.03–1.21)	0.004	1.09 (0.98–1.21)	0.079
Adolescence	Reference		Reference	
**Women occupation**				
Homemakers	Reference		Reference	
Labour	1.12 (0.98–1.27)	0.100	0.99 (0.85–1.17)	0.944
Business	1.31 (1.17–1.47)	< 0.001	1.41 (1.23–1.62)	< 0.001
Service Holder	1.36 (1.09–1.69)	0.005	1.33 (0.99–1.81)	0.061
**Total children ever born**				
≤ 2	Reference		Reference	
≥ 3	1.39 (1.23–1.49)	< 0.001	0.97 (0.88–1.06)	0.518
**Preceding birth interval (**years)				
< 2	0.82 (0.72–0.93)	0.003	0.82 (0.72–0.93)	0.002
2–4	Reference		Reference	
> 4	1.11 (1.01–1.21)	0.025	1.03 (0.94–1.13)	0.475
**Husband/partner’s current age (Years)**				
< 30	Reference		Reference	
30–34	1.78 (1.54–2.05)	< 0.001	1.36 (1.05–1.76)	0.022
35–39	2.21 (1.93–2.53)	< 0.001	1.39 (1.08–1.79)	0.011
≥ 40	2.65 (2.36–2.97)	< 0.001	1.38 (1.06–1.79)	0.015

Table 3 presents the decomposition of CCI concerning the wealth index, aiming to examine inequalities in PT. The decomposition analysis evaluates the contribution of various explanatory variables to the overall CCI, expressed as a percentage. The observed CCI for pregnancy termination is **0.029 (SE = 0.008**,**p < 0.001).** Our study found that adjusted percentage contribution of socioeconomic inequality in PT was largely driven by the wealth status (74.98%), mass media exposure (41.82%) and place of residence (34.35%). Furthermore, respondent’s occupations had a significant contribution to pregnancy termination related inequality (24.81%). Besides, the respondent’s preceding birth interval and husband/partner’s current age explained the percentage of the inequalities respectively about 7% and 6%.

**Table 3:** Decomposition of concentration index for measuring socioeconomic inequalities in pregnancy termination in Bangladesh

Study variables	Category	Elasticity	Concentration index	Contribution to overall CCI = 0.029 (*p* < 0.001)	
				Absolute contribution	Percentage contribution
**Administrative region**	Barisal	0.003	-0.133	-0.001	-1.344
	Chittagong	-0.042	0.129	-0.005	-18.878
	Dhaka	-0.027	0.453	-0.012	-41.574
	Khulna	Reference			
	Mymensingh	-0.014	-0.159	0.002	7.501
	Rajshahi	-0.012	-0.118	0.001	4.777
	Rangpur	0.007	-0.308	-0.002	-7.510
	Sylhet	-0.003	-0.032	0.00008	0.293
Subtotal				**-0.01692**	**-56.735**
**Place of residence**	Rural	Reference			
	Urban	0.032	0.312	0.010	34.353
Subtotal				**0.010**	**34.353**
**Religion**	Islam	Reference			
	Hinduism	-0.021	-0.089	0.002	6.439
	others	0.002	-0.142	-0.0002	-0.736
Subtotal				**0.0018**	**5.703**
**Sex of household head**	Male	Reference			
	Female	0.005	0.031	0.0001	0.486
Subtotal				**0.0001**	**0.486**
**Mass media exposure**	No	Reference			
	Yes	0.061	0.199	0.012	41.817
Subtotal				**0.012**	**41.817**
**Wealth index**	Poorest	0.008	-0.809	-0.006	-21.454
	Poorer	-0.034	-0.447	0.015	52.227
	Middle	Reference			
	Richer	-0.019	0.381	-0.007	-25.457
	Richest	0.027	0.757	0.020	69.660
Subtotal				**0.022**	**74.976**
**Women current age**	< 30 years	Reference			
	≥ 30 years	0.236	0.002	0.0004	1.494
Subtotal				**0.0004**	**1.494**
**Women height**	Below average (< 164 cm)	Reference			
	Average or above (≥ 164 cm)	-0.005	0.255	-0.001	-4.836
Subtotal				**-0.001**	**-4.836**
**Women age at first intercourse**	non-adolescence	Reference			
	adolescence	-0.007	0.171	-0.001	-4.291
Subtotal				**-0.001**	**-4.291**
**Women occupation**	Homemakers	Reference			
	Labor	0.033	0.067	0.002	7.730
	business	0.022	0.089	0.002	6.587
	Service Holder	0.007	0.445	0.003	10.488
Subtotal				**0.007**	**24.805**
**Wife beating**	low	Reference			
	high	0.020	-0.096	-0.002	-6.738
Subtotal				**-0.002**	**-6.738**
**Contraceptive usage**	No	Reference			
	Yes	0.014	-0.015	-0.0002	-0.758
Subtotal				**-0.0002**	**-0.758**
**Total children ever born**	≤ 2	Reference			
	≥ 3	0.020	-0.094	-0.002	-6.543
Subtotal				**-0.002**	**-6.543**
**Preceding birth interval**	< 2 years	-0.013	-0.042	0.0006	1.921
	2–4 years	Reference			
	> 4 years	0.035	0.038	0.001	4.612
Subtotal				**0.0016**	**6.533**
**Husband/partner’s current age (years)**	< 30	Reference			
	30–34	0.033	0.028	0.0009	3.192
	35–39	0.017	0.013	0.0002	0.756
	≥ 40	0.123	0.004	0.0005	1.658
Subtotal				**0.0016**	**5.606**
**Husband/partner’s occupation**	Unemployed	Reference			
	Farmer/Labor	0.058	-0.209	-0.012	-41.945
	Business	0.079	0.142	0.011	38.801
	Service Holder	0.004	0.449	0.002	6.394
Subtotal				**0.001**	**3.25**
Explained CI				**0.03238**	**111.962**
Residual CI				**-0.00338**	**11.962**

## Results from the measures of inequality

**Fig. 1:**
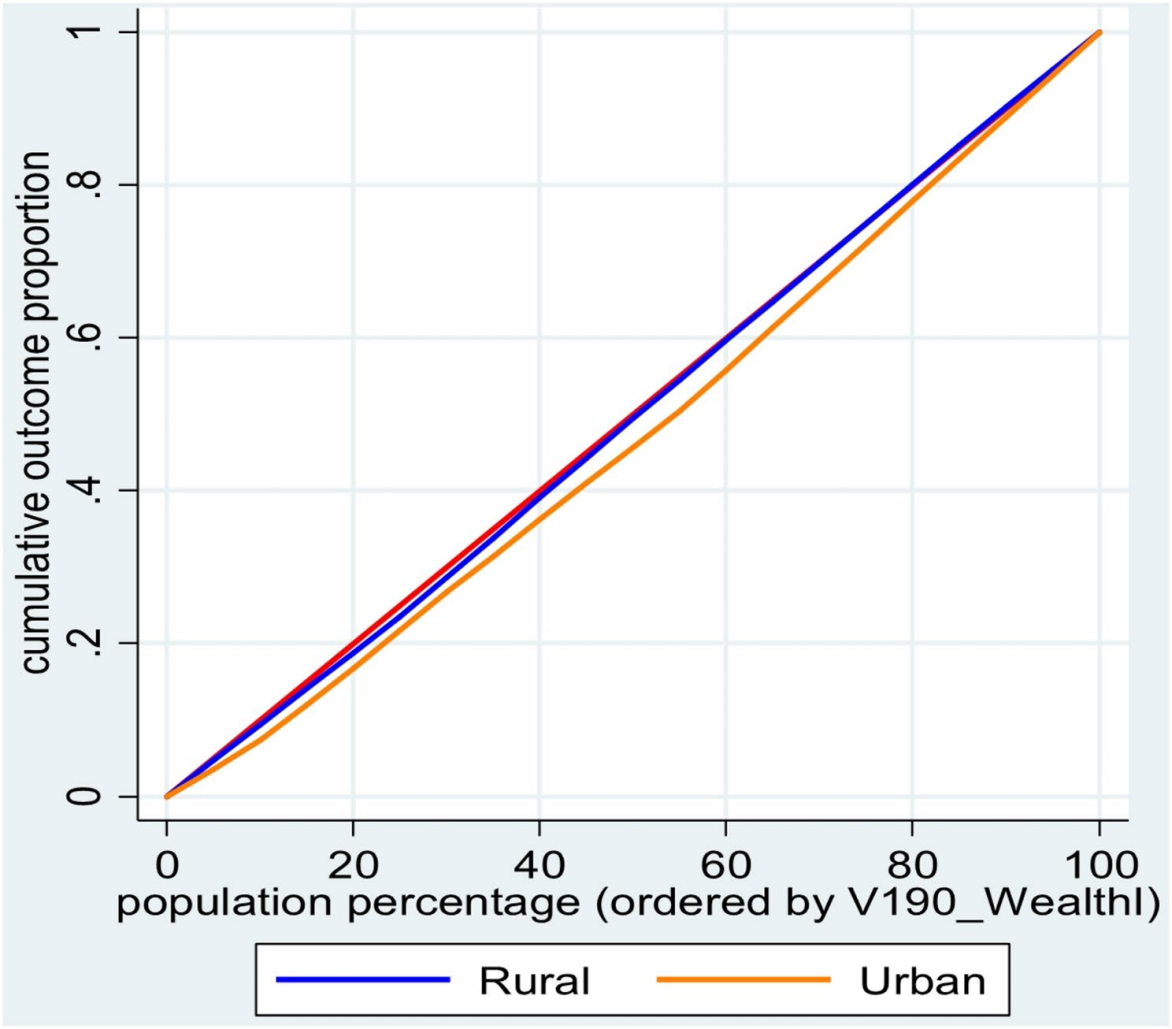
Concentration curve for inequality in pregnancy termination among women in Bangladesh and according to its place of residence

Figure 1 illustrates curves depicting the inequality in PT among Bangladesh residents. The concentration curve for terminated pregnancies in Bangladesh falls below the equity line, indicating a concentration of instances among women with higher wealth indices. Similarly, the concentration curve for residents of Bangladesh lies below the equality line, indicating a disproportionate prevalence of PT among urban women. The relative value of CCI for pregnancy termination, as shown in Table 3, is 0.029 (*p* < 0.001), signifying its significance. A positive CCI suggests that PT is more concentrated among wealthier women compared to poorer women.

## Discussion

This study analyzed data from the 2017-18 Bangladesh Demographic and Health Survey aimed to assess PT’s prevalence and socioeconomic disparities factors in Bangladesh. Findings showed that over 20% of ever-married women in Bangladesh have experienced pregnancy termination.

Our investigation revealed that women from the Chittagong and Sylhet divisions in Bangladesh were more likely to undergo PT compared to those from the Khulna division. This finding aligns with Zahan et al.‘s study, which identified areas like Panchagarh, Habiganj, and Sylhet as being at higher risk for PT [18]. Women who undergo PT often face increased restrictions and social stigma, consistent with previous research [19]. The relationship between PT and wealth index is complex [18]. Our study found a strong association between PT and the poorest wealth index, as well as an increased risk among women in the highest wealth quintile. This trend mirrors findings from studies on Nigerian and Ghanaian women, suggesting that the association between PT and wealth index may be influenced by women’s increased financial autonomy [20, 21]. Progressive women age (≥ 30 years) has a great effect on PT [22]. Our study revealed that women age ≥ 30 years is a significantly higher risk for terminated pregnancy in Bangladesh. Our result is reliable with US women [23]. Social support from family members, friends and partners are more advantageous for health of pregnant women [24].

Environmental factors in the workplace, such as extreme temperatures, can interfere with fetal development, potentially leading to fetal anomalies among pregnant women [25]. Our analysis found a significant association between businesswomen and the risk of PT, consistent with a study on Ghanaian women based on Ghana DHS data [21]. Workplace environments that support pregnant women may help reduce the risk of PT [18]. Birth spacing is crucial in obstetric practice [26], and our study revealed a significant link between short birth intervals (< 2 years) and PT. Mothers with short birth intervals were found to have higher odds of PT compared to their counterparts, aligning with studies conducted in 36 sub-Saharan African countries [27]. A Kenyan study suggests a minimum of 2 years for the complete restoration of women’s nutritional and physical status depleted from the prior pregnancy [28]. Increasing paternal age and paternally inherited genetic mutations in the embryo can contribute to terminated pregnancies [29]. Our study found significant associations between the husband/partner’s current age groups (30–34 years, 35–39 years, and ≥ 40 years) and PT, consistent with findings from studies conducted in France and California, USA [30, 31].

Socioeconomic inequality is a key public health anxiety [32]. Socioeconomic position of women which are interpreted as unequal opportunities for choosing the pregnancy outcome [33]. Our decomposition analysis depicts that wealthier household women were the most significant factor that indicates pro-rich inequality of pregnancy termination in Bangladesh. Our study is consistent with Turkish women’s study [34]. Women from richer households are more willing to PT comparing poorer women. Rich women, particularly women from the rich wealth index should be careful to avoid unhealthy lifestyles to prevent PT. The decomposition results also show that usage of mass media exposure and place of residence contributed to the increase of socioeconomic inequality in pregnancy termination during the survey time [35, 36]. Richer wealth index women were classically busy with their occupation which contributed to the experiential increase in inequality of pregnancy termination. Besides, the respondent’s husband’s age explained the escalation of the inequality of pregnancy termination [37].

Furthermore, it is worth noting that inequality of access to contraception, not only in the developing and underdeveloped countries but also in the developed countries. Internal country policies and the absence of clear scientific guidelines can lead to significant inequalities with a consequent impact on the incidence of PT in women at risk [38–41].

### Policy implications

Our study proposes strategic measures to prevent PT among working pregnant women in Bangladesh. Firstly, enforcing maternity leave regulations and improving workplace conditions could offer significant support. Secondly, promoting healthy family planning and fostering better conjugal relationships may enhance women’s reproductive health outcomes. Lastly, mapping the risk of PT across different regions can aid in identifying high-risk areas for targeted interventions.

### Strength and limitations

The study’s strengths lie in its use of a nationally representative sample of women respondents and the adoption of validated surveys like the DHS, bolstering the robustness of our findings. We emphasized the importance of valid statistical inferences for continuous covariates and highlighted the potential benefits of targeting high-risk regions for reproductive health interventions. Additionally, the use of decomposition analysis a widely accepted method in public health inequality measurement enabled us to quantify the contributions of various factors to PT outcomes.

However, our study also had limitations. Firstly, the use of cross-sectional secondary data limits the scope of our analysis. Secondly, our analysis only considered existing variables in the BDHS data, neglecting potential influences of various genetic hormones, which might also be associated with PT.

## Conclusions

PT is a great issue of public health for life-threatening. The findings showed that wealth index, administrative region, women’s occupation, birth interval, and husband/partner’s current age are factors associated with PT. Implementation of integrated community-based programs for effective contraception can generate awareness and reduce PT rates. Additionally, media campaigns can play a vital role in reducing the growing burden of PT by disseminating information and promoting responsible reproductive health practices.

## Data Availability

The datasets used and/or analyzed during the current study are available from the corresponding author on reasonable request.
